# Tension-dependent removal of pericentromeric shugoshin is an indicator of sister chromosome biorientation

**DOI:** 10.1101/gad.240291.114

**Published:** 2014-06-15

**Authors:** Olga O. Nerusheva, Stefan Galander, Josefin Fernius, David Kelly, Adele L. Marston

**Affiliations:** The Wellcome Trust Centre for Cell Biology, School of Biological Sciences, University of Edinburgh, Edinburgh EH9 3JR, United Kingdom

**Keywords:** mitosis, meiosis, shugoshin, biorientation, tension, kinetochore

## Abstract

During mitosis and meiosis, sister chromatid cohesion resists the pulling forces of microtubules, enabling the generation of tension at kinetochores upon chromosome biorientation. How tension is read to signal the bioriented state remains unclear. Shugoshins form a pericentromeric platform that integrates multiple functions to ensure proper chromosome biorientation. Here we show that budding yeast shugoshin Sgo1 dissociates from the pericentromere reversibly in response to tension. The antagonistic activities of the kinetochore-associated Bub1 kinase and the Sgo1-bound phosphatase protein phosphatase 2A (PP2A)-Rts1 underlie a tension-dependent circuitry that enables Sgo1 removal upon sister kinetochore biorientation. Sgo1 dissociation from the pericentromere triggers dissociation of condensin and Aurora B from the centromere, thereby stabilizing the bioriented state. Conversely, forcing sister kinetochores to be under tension during meiosis I leads to premature Sgo1 removal and precocious loss of pericentromeric cohesion. Overall, we show that the pivotal role of shugoshin is to build a platform at the pericentromere that attracts activities that respond to the absence of tension between sister kinetochores. Disassembly of this platform in response to intersister kinetochore tension signals the bioriented state. Therefore, tension sensing by shugoshin is a central mechanism by which the bioriented state is read.

## Abstract (precis)

This study addresses how tension between chromosomes signals their proper alignment for segregation during cell division. Marston and colleagues show that budding yeast shugoshin dissociates from the pericentromere reversibly in response to tension. The antagonistic activities of the kinetochore-associated Bub1 kinase and the shugoshin-bound phosphatase PP2A-Rts1 underlie a tension-dependent circuitry that enables shugoshin removal upon sister kinetochore biorientation. The findings expose shugoshin as the pivotal sensor of tension between sister kinetochores.

For accurate dissemination of the genome, chromosomes are first duplicated during S phase of the cell cycle to generate identical sister chromatids that are held together by cohesion. During mitosis, to ensure that each daughter cell receives one copy of each chromosome after cohesion is lost, sister kinetochores must attach to microtubules from opposite poles. Cohesion resists the pulling force of microtubules, resulting in the generation of tension at sister kinetochores. Sister kinetochore tension is critical in enabling biorientation to be sensed, thereby allowing chromosome segregation to proceed. Although central to the segregation process, the underlying mechanism by which this state of tension is read is not known.

The conserved shugoshin family of proteins has been implicated in the sensing of intersister kinetochore tension (Indjeian et al. 2005; Huang et al. 2007; Kiburz et al. 2008; Kawashima et al. 2007; Vanoosthuyse et al. 2007). Shugoshins are localized to the region surrounding the centromere (the pericentromere) in a manner dependent on the cohesin complex and phosphorylation of histone 2A on residue S121 by the kinetochore-associated kinase Bub1 (Kawashima et al. 2010; Liu et al. 2013a). Shugoshins are emerging as important pericentromeric “adaptor” proteins that integrate multiple functions that contribute to accurate chromosome segregation (Gutiérrez-Caballero et al. 2012; Rattani et al. 2013; Verzijlbergen et al. 2014). Shugoshins were first identified as regulators of chromosome segregation during meiosis (Katis et al. 2004; Kitajima et al. 2004; Marston et al. 2004; Rabitsch et al. 2004; Kerrebrock et al. 1992). During meiosis I, a unique segregation event occurs in which the maternal and paternal chromosomes (homologs) are separated and that requires homologs to be linked, usually by chiasmata, the products of meiotic recombination (for reviews, see Marston and Amon 2004; Marston 2014). Also during meiosis I, sister kinetochore biorientation is suppressed, and sister kinetochores attach to microtubules from the same pole, known as mono-orientation, to ensure the cosegregation of sister chromatids. Once homologs are aligned on the meiotic spindle, cohesion is lost from chromosome arms, resolving chiasmata and triggering the segregation of homologs. However, cohesion in the pericentromere must be protected from loss during meiosis I to allow the biorientation of sister chromatids during meiosis II. The protection of pericentromeric cohesin during meiosis I depends on shugoshin, which recruits protein phosphatase 2A associated with its B′-type regulatory subunit (PP2A-B′) to the pericentromere (Kitajima et al. 2006; Riedel et al. 2006). PP2A-B′ reverses phosphorylation of the meiosis-specific Rec8 subunit of cohesin in the pericentromere, making it refractory to cleavage by the protease separase (Brar et al. 2006; Ishiguro et al. 2010; Katis et al. 2010; Attner et al. 2013). Shugoshin similarly spatially regulates cohesin loss during mammalian mitosis, where the bulk of cohesin dissociates from chromosome arms during prophase due to the activity of the destabilizing protein Wapl (Waizenegger et al. 2000; Hauf et al. 2005; Kueng et al. 2006; Tang et al. 2006; Shintomi and Hirano 2009). In this case, the shugoshin–PP2A-B′ complex dephosphorylates the Wapl-counteracting protein sororin, thereby maintaining its pericentromeric localization (Nishiyama et al. 2010; Liu et al. 2013b).

In addition to protecting pericentromeric cohesin during meiosis and mammalian mitosis, shugoshins play a conserved role in promoting biorientation of sister chromatids (Indjeian et al. 2005; Huang et al. 2007; Kiburz et al. 2008). Biorientation is achieved owing to a bias for sister kinetochores to be captured from opposite poles together with an error correction mechanism that destabilizes incorrect attachments that lack tension (for review, see Tanaka 2010). We recently found that shugoshins contribute to sister kinetochore biorientation by both enabling the bias to capture by microtubules from opposite poles and engaging the error correction machinery (Verzijlbergen et al. 2014). Error correction relies on the chromosomal passenger complex (CPC), which is comprised of Aurora B kinase (Ipl1 in budding yeast) and its centromere targeting factor, survivin (Bir1), together with INCENP (Sli15) and borealin (Nbl1) (for review, see Carmena et al. 2012). Maintenance of the CPC at centromeres requires shugoshin (Kawashima et al. 2007; Vanoosthuyse et al. 2007; Yamagishi et al. 2010; Rivera et al. 2012; Verzijlbergen et al. 2014). Additionally, shugoshin recruits the chromosome-organizing complex condensin to the pericentromere to bias sister kinetochores toward biorientation (Verzijlbergen et al. 2014). Therefore, overall, Shugoshin acts as an adaptor that attracts multiple activities, including PP2A-B′, CPC, and condensin, to the pericentromere to safeguard accurate chromosome segregation.

Although the ability to discriminate between tension-generating and tension-less attachments is the key to achieving chromosome biorientation (Nicklas and Ward 1994), it is not well understood. One way in which changes in kinetochore tension can be sensed is distance-dependent substrate accessibility (for review, see Lampson and Cheeseman 2011). Indeed, in the absence of tension, the outer kinetochore and the inner centromere (where the CPC is localized) are in close proximity. In contrast, tension moves the outer kinetochore away from the inner centromere. This spatial separation is thought to allow outer kinetochore substrates to evade the reach of Aurora B phosphorylation, thereby stabilizing kinetochore–microtubule attachments (Keating et al. 2009; Liu et al. 2009; Welburn et al. 2010). However, this model has recently been challenged by the finding that the centromere localization of the CPC does not need to be tightly regulated for tension sensing by Aurora B in budding yeast, suggesting that other mechanisms may contribute (Campbell and Desai 2013).

Interestingly, in both mitosis and meiosis, shugoshin plays its critical roles at pericentromeres only when sister kinetochores are not under tension. This suggests that shugoshins may govern the tension-sensing process. Indeed, shugoshin undergoes a tension-dependent relocation from the inner centromere to the kinetochores in spermatocytes, oocytes, and human somatic cells (Gomez et al. 2007; Lee et al. 2008; Liu et al. 2013a). In human cells, the tension-dependent relocalization of shugoshin to kinetochores is triggered by dephosphorylation and is important for accurate segregation (Liu et al. 2013a). However the underlying mechanism of this relocation and its role is not well understood. Here we use budding yeast to address the role of spindle tension in the regulation and function of its single shugoshin, Sgo1. We show that intersister kinetochore tension negatively regulates Sgo1 association with pericentromeric chromatin. Spatial separation of the kinetochore-associated Bub1 kinase triggers Sgo1 removal from the pericentromere, facilitated by Sgo1 association with PP2A. We further show that Sgo1 release from the pericentromere triggers Aurora B removal upon biorientation, thereby initiating the silencing of the error correction process. Finally, we demonstrate that the protection of pericentromeric cohesin in meiosis I by Sgo1 relies on the suppression of sister kinetochore biorientation. Overall, our findings reveal tension-dependent Sgo1 removal from the pericentromere as a fundamental sign that a pair of sister kinetochores has bioriented.

## Results

### Spindle tension between sister kinetochores promotes Sgo1 removal from the pericentromere during mitosis

**Figure 1.**
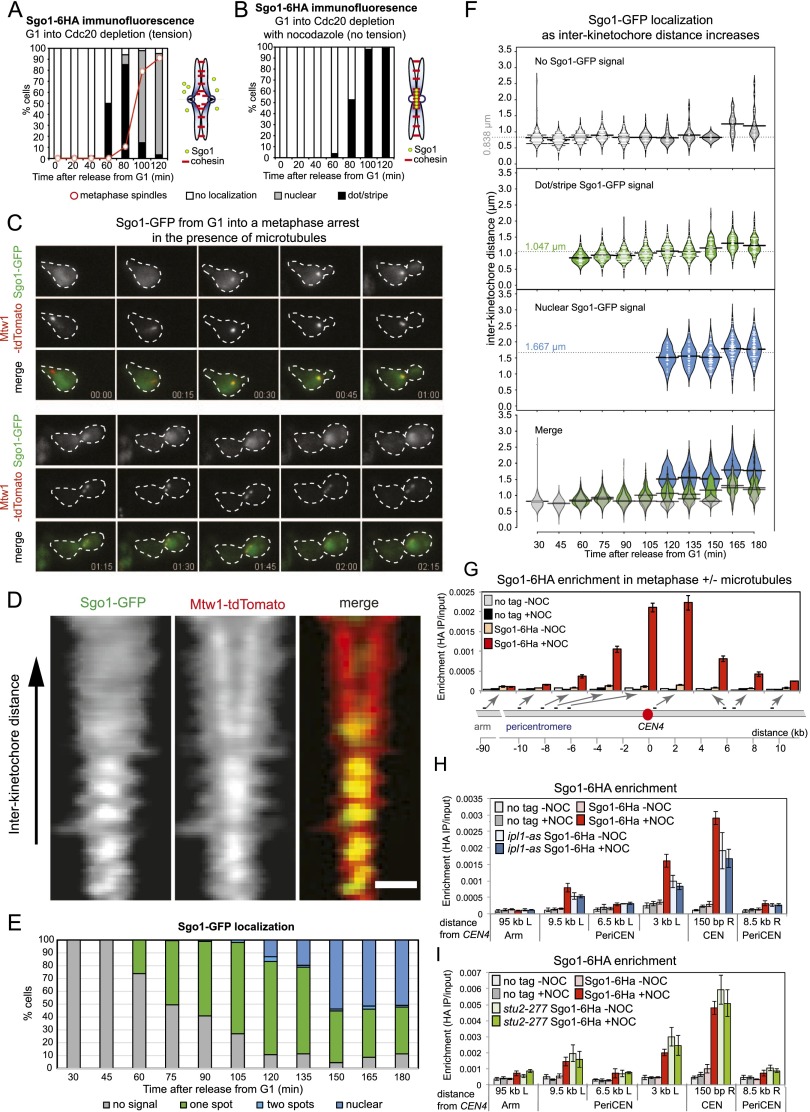
Sgo1 is removed from the pericentromere in metaphase in the presence of microtubules. (*A*,*B*) Sgo1 dispersal into the nucleus in metaphase is dependent on microtubules. Cells carrying *SGO1-6HA* and *pMET3-CDC20* (strain AM6390) were arrested in G1 with α factor. The culture was split, α factor was washed out, and both cultures were released into medium containing methionine to repress *CDC20* and induce arrest in metaphase. Either DMSO (*A*; tension) or nocodazole (*B*; no tension) was added. Samples were extracted at the indicated intervals after release from G1 for Sgo1-6HA and tubulin immunofluorescence, and Sgo1 localization (no, dot/stripe, nuclear) and spindle morphology were scored. Schematic diagrams indicate chromosome configuration in the presence (*A*) or absence (*B*) of tension. (*C*) Loss of Sgo1-yeGFP from the pericentromere coincides with the appearance of a bilobed kinetochore signal. Cells carrying *SGO1-yeGFP* and *MTW1-tdTomato* (strain AM9233) were imaged on a microfluidics device at 15-min intervals after release from G1 arrest. (*D–F*) Sgo1-yeGFP loses its pericentromeric localization as kinetochore signals split. Strain AM9233 (*pMET3-CDC20 SGO1-yeGFP MTW1-tdTomato*) was arrested in G1 using α factor and released in medium containing 8 mM methionine to deplete Cdc20. Images of multiple cells were taken every 15 min, with the first time point taken 0.5 h after the release from G1. (*D*) Line scans across kinetochore foci of single cells were assembled from 100 images to generate a V plot showing Sgo1-GFP localization as interkinetochore distance increases. Bar, 2μm. (*E*) Bar chart showing the fraction of cells with the indicated Sgo1 localization at each time point. (*F*) The distance between Mtw1-tdTomato signals and the localization of Sgo1-yeGFP was scored in 200 cells. The bean plot shows the distribution of interkinetochore distances for which each localization type was scored. The horizontal line represents the mean. (*G*) Sgo1 is removed from the pericentromere at metaphase in the presence of microtubules. Strains AM6390 (*pMET3-CDC20 SGO1-6HA*) and AM2508 (*pMET3-CDC20*; no tag control) were released from G1 into medium containing methionine and either DMSO (−NOC) or nocodazole (+NOC). After 2 h, cells were harvested, and Sgo1-6HA levels at the indicated sites on chromosome IV were analyzed by ChIP-qPCR. The average of three experimental repeats (qPCR performed in triplicate in each case) is shown for AM6390, with error bars representing standard error. For the no tag control (AM2508), representative values are shown from one of these experiments. See also Supplemental Figure S2, G and H, for Sgo1-6HA association with sites on chromosomes III and V. (*H*) Wild-type (AM6390) and *ipl1-as5* (AM8217) cells carrying *pMET3-CDC20* and *SGO1-6HA* as well as a no tag control (AM2508) were treated as in *G* except that NA-PP1 (50 mM) was added to inhibit Ipl1 when bud formation was observed after release from G1. Sgo1-6HA levels at the indicated sites on chromosome IV were measured by ChIP-qPCR in cells harvested 2 h (wild type) or 2.5 h (*ipl1-as*) after release from G1 to obtain a similar number of cells arrested in metaphase. (*I*) The *stu2-277* mutation prevents Sgo1 removal in the presence of microtubules. Wild-type (AM6390) and *stu2-277* (AM9093) cells carrying *pMET3-CDC20* and *SGO1-6HA* as well as a no tag control (AM2508) were treated as in *G* except that cells were shifted to 37°C after release from G1. Cells were harvested for Sgo1-6HA ChIP-qPCR after 1.5 h (wild type) or 2.25 h (*stu2-277*) to obtain similar numbers of cells arrested in metaphase. In *H* and *I*, the average of three independent repeats is shown, with error bars representing standard error.

Budding yeast have a single shugoshin protein, Sgo1, that localizes to the pericentromere and functions to both protect cohesin in meiosis I and promote sister kinetochore biorientation in mitosis. To explore the possibility that intersister kinetochore tension regulates Sgo1 distribution, we monitored Sgo1-6HA localization by immunofluorescence as cells progressed from G1 into a metaphase arrest in either the presence or absence of microtubules. We used cells in which the essential APC regulator *CDC20* was placed under the control of the methionine-repressible promoter *pMET3* (*pMET3-CDC20*) to induce metaphase arrest by addition of methionine. Cells carrying *SGO1-6HA* and *pMET3-CDC20* were released from G1 into medium containing methionine and either nocodazole (to depolymerize microtubules) or DMSO (as a control). In cells that were not treated with nocodazole, Sgo1 first appeared as a bright dot within the nucleus, likely representing the pericentromere (Kiburz et al. 2005). Interestingly, by 100 min after release from G1, the Sgo1-GFP signal had dissipated throughout the nucleus (Fig. 1A). However, in nocodazole-treated cells, the dot-like Sgo1-6HA localization persisted, and uniform nuclear staining was not observed (Fig. 1B). Consistently, treatment of live cells with increasing doses of microtubule-depolymerizing drugs was shown to increase Sgo1 levels at the pericentromere (Haase et al. 2012). These findings suggest that metaphase spindle formation triggers the release of Sgo1-6HA from the pericentromere into the nucleus.

Sgo1 is absent in α-factor-arrested G1 cells, accumulates upon cell cycle entry, and is degraded during anaphase (Marston et al. 2004). In cells released from a G1 arrest, chromatin immunoprecipitation (ChIP) showed that Sgo1 associates with the pericentromere and is later dispersed into the nucleus prior to its degradation in anaphase, demonstrating that release from the pericentromere is not a consequence of the metaphase arrest (Supplemental Fig. S1A–G).

### Sgo1 dispersal into the nucleus occurs as sister kinetochores biorient

To more accurately determine the relative timing of the establishment of intersister kinetochore tension and Sgo1 removal from the pericentromere, we released live cells with labeled kinetochores (*MTW1-tdTomato*) and *SGO1-GFP* from a G1 arrest and imaged them at 15-min intervals as they progressed into a metaphase arrest induced by *CDC20* depletion (Fig. 1C; Supplemental Movie S1). This confirmed that Sgo1 initially appears as a bright pericentromeric dot before dispersing into the nucleus during metaphase (Fig. 1C; Supplemental Movie S1), and this was also observed in cells that were not arrested in metaphase or previously arrested in G1 (Supplemental Fig. S1H,I). Fluorescence intensity measurements confirmed depletion of Sgo1-GFP from the area occupied by the kinetochores and spindle during metaphase (Supplemental Fig. S1J,K). Assembled line scans of kinetochore foci separated by increasing distance suggested that Sgo1 release from the pericentromere correlated with increased interkinetochore distance (Fig. 1D). We measured the longest distance covered by the Mtw1-tdTomato foci and scored the Sgo1-GFP signal in at least 200 live cells at 15-min intervals after release from G1. Figure 1, E and F, shows that release of Sgo1-GFP into the nucleus occurred as Mtw1-tdTomato distance increased to ∼1.5 μm (120 min after release from G1). Therefore, Sgo1 removal from the pericentromere occurs concomitant with the establishment of intersister kinetochore tension and biorientation.

### Sgo1 is absent from pericentromeres under tension

To test whether the disappearance of the subnuclear Sgo1-GFP dot upon tension establishment corresponds to Sgo1 release from the pericentromeric chromatin, we sought to use ChIP. Based on ChIP assays, the localization of cohesin and its Scc2 loader in the pericentromere is thought to be negatively regulated by tension (Eckert et al. 2007; Ocampo-Hafalla et al. 2007; Kogut et al. 2009). Indeed, the recovery of pericentromeric sequences after ChIP of the cohesin subunit Scc1 is lower when cells are arrested in metaphase with microtubules compared with those without microtubules (Supplemental Fig. S2A–C; Eckert et al. 2007; Ocampo-Hafalla et al. 2007; Kogut et al. 2009). However, live-cell microscopy experiments have shown that cohesin remains localized at pericentromeres during metaphase, questioning the significance of the ChIP experiments (Mc Intyre et al. 2007; Yeh et al. 2008; Rowland et al. 2009). Indeed, we found that centromeric quantitative PCR (qPCR) values were also reduced by the presence of microtubules when the constitutive kinetochore subunits Mtw1 and Ndc10 were immunoprecipitated (Supplemental Fig. S2D,E). Moreover, the levels of TetR-GFP artificially tethered to *tetO*s adjacent to *CEN3* were also reduced twofold by the presence of microtubules as measured by ChIP (Supplemental Fig. S2F). It is unlikely that tension causes removal of core kinetochore proteins and tethered TetR-GFP from the centromere, as no such change was observed by microscopy (e.g., Fig. 1C; OO Nerusheva and AL Marston, unpubl.). Instead, we suggest that the difference relates to a reduced ChIP efficiency of pericentromeric sequences separated by tension. Importantly, only where the pericentromeric ChIP-qPCR signal is reduced more than twofold by microtubule-dependent tension can we be confident that this is due to a decrease in the association of the protein measured.

With this in mind, we used ChIP to analyze Sgo1 association with the pericentromere in cells arrested in metaphase in the presence and absence of microtubules. Cells carrying *pMET3-CDC20* and *SGO1-6HA* as well as a no tag control were treated with methionine to induce a metaphase arrest in either the presence or absence of nocodazole, and Sgo1-6HA levels were analyzed by ChIP-qPCR on chromosomes III, IV, and V (Fig. 1G; Supplemental Fig. S2G,H). Spindle length measurements and Pds1 staining confirmed that the majority of cells remained arrested in metaphase at the time of harvesting (Supplemental Fig. 2I,J). As our live-cell analysis revealed, Sgo1 associates with the pericentromere only when sister kinetochores are not under tension (Fig. 1G; Supplemental Fig. S2G,H). Sgo1 association with the pericentromere is not dependent on spindle checkpoint activation in response to unattached kinetochores generated by the nocodazole treatment because deletion of the spindle checkpoint component *MAD2* did not reduce Sgo1 protein levels or its association with the pericentromere (Supplemental Fig. S2K,L). Addition of nocodazole to cells already arrested in metaphase led to Sgo1 accumulation at the centromere, indicating that Sgo1 removal under tension is reversible (Supplemental Fig. S2M,N). We conclude that Sgo1 associates with the pericentromere only in the absence of microtubules and that this reduction can be readily observed by ChIP.

### Intersister kinetochore tension is responsible for Sgo1 removal from the pericentromere

Our findings suggest that Sgo1 association with the pericentromere is negatively regulated by microtubules. To further investigate the effect of tension on Sgo1 removal, we employed two methods that reduce kinetochore tension. First, we inactivated the Aurora B kinase (Ipl1) using a version (*ipl1-as5*) sensitive to the ATP analog NAPP1, which results in syntelic attachments (both sister kinetochores attached to microtubules from the same pole) (Fig. 1H; Pinsky et al. 2006b). Although reduced compared with the wild type, Sgo1-6HA ChIP-qPCR values were similar in NAPP1-treated *ipl1-as* metaphase-arrested cells in the presence and absence of microtubules. Although we cannot rule out a direct effect of Ipl1, which is known to associate with Sgo1 (Verzijlbergen et al. 2014), this finding supports the idea that intersister kinetochore tension triggers Sgo1 removal from the pericentromere. As an alternative way to abolish intersister kinetochore tension while kinetochores are attached to microtubules, we used a strain in which the function of the microtubule assembly protein Stu2 (ortholog of XMAP215/Dis1) is impaired. Strains harboring the *stu2-277* allele grown at the restrictive temperature have reduced microtubule dynamics, resulting in prevalent monotelic and syntelic attachments (He et al. 2001; Pearson et al. 2003; Marco et al. 2013). In *stu2-277* metaphase-arrested cells, similar levels of Sgo1-6HA were associated with the pericentromere in both the presence and absence of nocodazole (Fig. 1I). The observation that Sgo1 is not removed from the pericentromere in the presence of microtubules either upon Ipl1 inhibition or in the presence of the *stu2-227* allele, both of which reduce tension, is strong support for the idea that intersister kinetochore tension triggers Sgo1 removal from the pericentromere.

### Biorientation of sister kinetochores removes Bub1 from the centromere

**Figure 2.**
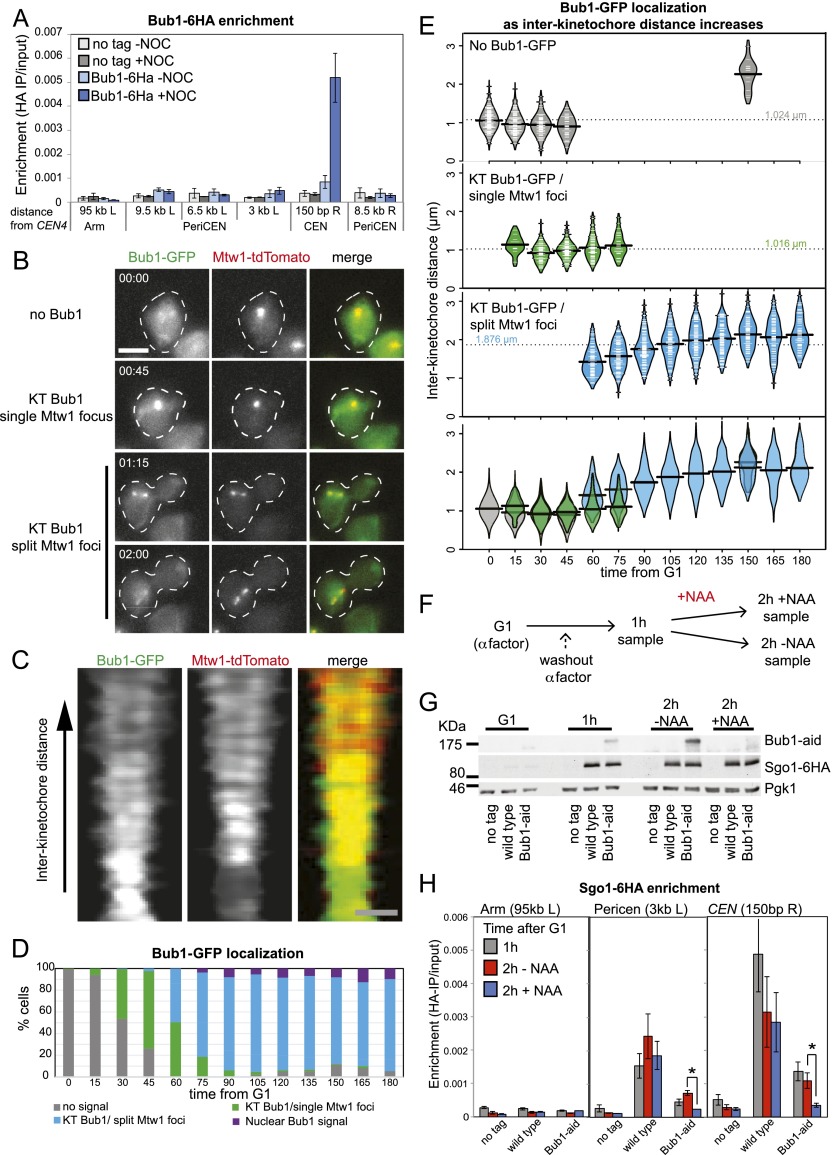
Bub1 is removed from kinetochores later than Sgo1 dissociates from the pericentromere. (*A*) Bub1 associates with centromeres in metaphase-arrested cells only in the absence of spindle tension. Cells (strain AM7449) carrying *BUB1-6HA* and *pMET3-CDC20* and a no tag control (AM2508) were treated as described in Figure 1G. Bub1-6HA levels at the indicated sites were measured by ChIP-qPCR. The average of three experimental repeats is shown, with error bars representing standard error. (*B–E*) Bub1 is retained at kinetochores upon separation of kinetochore clusters. Cells carrying *BUB1-yeGFP* and *MTW1-tdTomato* (strain AM9229) were imaged on a microfluidics device at 15-min intervals after release from G1 arrest. (*B*) Cells exhibiting different types of Bub1-GFP localization at the indicated time points are shown. Bar, 5 μm. (*C*) Line scans across kinetochore foci of single cells were assembled from 100 images to generate a V plot showing Bub1-yeGFP localization as interkinetochore distance increases. Bar, 2 μm. (*D*) Bar chart with the fraction of cells with the indicated Bub1 localization at each time point is shown. (*E*) The distance between Mtw1-tdTomato signals and the localization of Bub1-yeGFP was scored in at least 90 cells for each time point. The bean plot shows the distribution of interkinetochore distances for which each localization type was scored. Lines within the beans represent individual cells. Beans for small sets of cells (*N* < 10) are not shown. The horizontal line represents the mean. (*F–H*) Continued Bub1 presence at kinetochores is required for Sgo1 localization at the pericentromere. (*F*) Scheme of the experiment is shown. Wild-type (AM6390) and *bub1-aid OsTir1* (AM9096) cells carrying *SGO1-6HA* and a no tag control (AM2508), all carrying *pMET3-CDC20*, were released from G1 into methionine and nocodazole-containing medium. After 1 h, one-third of the culture was harvested for ChIP and Western blotting, the remaining culture was split, and NAA was added to one half. After 2 h total, the remaining cultures were harvested. (*G*) Western immunoblot analysis was performed with anti-aid, anti-HA, and anti-Pgk1 antibodies to confirm that Bub1 is degraded upon NAA treatment, but Sgo1 is not. Pgk1 is shown as a loading control. (*H*) ChIP-qPCR analysis of Sgo1 localization at the indicated sites on chromosome IV. The mean of three experimental repeats is shown, with error bars indicating standard error. Student’s *t*-test was used to calculate confidence values. (*) *P* < 0.05.

Sgo1 association with the pericentromere depends on the Bub1 kinase (Fernius and Hardwick 2007). Bub1-dependent phosphorylation of histone H2A on Ser121 (H2A-S121) is important for Sgo1 recruitment to the pericentromere (Kawashima et al. 2010). Bub1 is positioned closer to the inner centromere than Sgo1 (Haase et al. 2012) but is also released from kinetochores as mitosis proceeds (Gillett et al. 2004). To test the idea that tension may also regulate Bub1 localization, we used ChIP-qPCR in cells arrested in metaphase in both the presence and absence of microtubules. Bub1-6HA was restricted to the core centromere, as expected, but is localized only in the absence of microtubules (Fig. 2A). This indicates that, like Sgo1, Bub1 is distant from the chromatin when sister kinetochores are under tension. We used live cells carrying *BUB1-GFP* and *MTW1-tdTomato* to correlate Bub1 disappearance with sister kinetochore separation (Fig. 2B–E; Supplemental Movie S2). Bub1-GFP colocalized with the kinetochore cluster soon after release from G1. As the Mtw1-tdTomato signal became bilobed, two Bub1-GFP foci were observed (Fig. 2B,C). Indeed, Bub1-GFP colocalized with kinetochore clusters separated to distances of >1.5 μm (Fig. 2D,E), where Sgo1-GFP was predominantly nuclear (Fig. 1F). This is consistent with a previous report that Bub1 and Sgo1 are spatially separated at metaphase (Haase et al. 2012). Together, our observations indicate that either Bub1 removal from kinetochores is not the trigger for Sgo1-GFP release from the pericentromere or kinetochore stretching upon tension is sufficient to move Bub1 away from substrates important for Sgo1 localization.

### Bub1 removal is sufficient for Sgo1 removal

To determine whether the continued presence of Bub1 is essential for the maintenance of Sgo1 in the pericentromere, we used the auxin-inducible degron (aid) system (Nishimura et al. 2009) to conditionally degrade Bub1 in metaphase-arrested cells in the presence of nocodazole. Cells were harvested 1 h after release from G1 into nocodazole, and Sgo1 levels were measured by ChIP-qPCR. Subsequently, the culture was split: One-half of the culture was treated with auxin (NAA) to induce Bub1 degradation, while the other half received no NAA (Fig. 2F). Prior to Bub1 degradation, as expected, Sgo1 was localized throughout the pericentromere, although levels in the Bub1-aid strain were considerably lower, presumably due to the partial functionality or stability of the Bub1-aid fusion protein (Fig. 2H). However, addition of auxin (NAA) led to Bub1 degradation, and Sgo1 was delocalized from the pericentromere (Fig. 2G,H). We conclude that continued Bub1 presence is required for Sgo1 maintenance at the pericentromere.

### Sgo1 is increased at the pericentromere in the absence of Rts1

**Figure 3.**
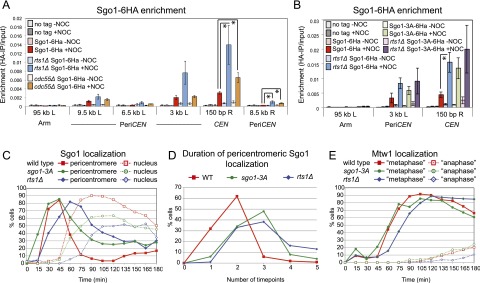
Association with PP2A^Rts1^ is required for timely Sgo1 removal from the pericentromere. (*A*) Pericentromeric Sgo1 levels are regulated by Rts1 and Cdc55. Wild-type (AM6390), *rts1Δ* (AM8859), and *cdc55Δ* (AM8957) cells carrying *SGO1-6HA* and *pMET3-CDC20* and a no tag *pMET3-CDC20* control (AM2508) were arrested in metaphase in the presence or absence of microtubules as described in Figure 1G, and anti-HA ChIP was performed followed by qPCR with primer sets at the indicated locations on chromosome IV. The average of four experimental repeats is shown, with error bars representing standard error. Student’s *t*-test was used to calculate confidence values. (*) *P* < 0.05. (*B*) Interaction with PP2A is required to control Sgo1 levels on the centromere. Wild-type and *rts1Δ* cells carrying *SGO1-6HA* (AM6390 and AM8859) or *SGO1-3A-6HA* (AM10143 and AM11902) and *pMET3-CDC20* together with a no tag control (AM2508) were grown and processed for ChIP-qPCR as described in *A*. The average of three experimental replicates are shown, with error bars representing standard error. (*C*,*D*) Sgo1 removal from the pericentromere is delayed in the absence of associated PP2A^Rts1^. Wild-type (AM9233) or *rts1Δ* (AM9735) cells producing *SGO1-yeGFP* and *SGO1-3A-yeGFP* (AM9873) cells, all carrying *pMET3-CDC20* and *MTW1-tdTomato*, were released from a G1 arrest on a microfluidics plate, and images were grabbed every 15 min. (*C*) Sgo1 localization was scored in at least 150 cells from each time point. (*D*) The number of frames in which pericentromeric Sgo1 signal was observed was scored for 100 cells per strain. (*E*) Bilobed Mtw1-tdTomato signal was scored in at least 150 cells as a marker of cell cycle progression.

The finding that inactivation of Bub1 kinase leads to Sgo1 removal from the pericentromere predicts the existence of a phosphatase that reverses Bub1-dependent phosphorylation. A prime candidate is the PP2A, a tripartite enzyme comprised of a scaffold (A), regulatory (B), and catalytic (C) subunit (Shi 2009). In budding yeast, there are two alternative regulatory subunits, Rts1 and Cdc55. PP2A-Rts1 associates with Sgo1 during mitosis and meiosis (Riedel et al. 2006; Xu et al. 2009), whereas PP2A-Cdc55 acts downstream from Sgo1 in preventing anaphase onset (Clift et al. 2009; Bizzari and Marston 2011; Yaakov et al. 2012). We examined the levels of Sgo1 at the pericentromere in cells lacking the PP2A regulatory subunits Rts1 or Cdc55. While pericentromeric levels of Sgo1 were modestly increased in *cdc55Δ* cells arrested in metaphase without microtubules, deletion of *RTS1* led to an approximately fourfold increase in pericentromeric Sgo1, although total cellular levels remained unchanged (Fig. 3A; Supplemental Fig. S3A). However, the majority of Sgo1 was removed when sister kinetochores were under tension even in cells lacking *RTS1* (Fig. 3A). This suggests that PP2A-Rts1 plays the predominant role in reducing Sgo1 levels at the pericentromere, with other phosphatases, including PP2A-Cdc55, also being important.

To determine whether direct association with PP2A is important for controlling the pericentromeric levels of Sgo1, we analyzed the *sgo1-3A* mutant, which fails to associate with PP2A (Xu et al. 2009). Similar to wild-type Sgo1 in cells lacking *RTS1*, the levels of the mutant Sgo1-3A protein were not increased overall (Supplemental Fig. S3B), accumulated to high levels on the pericentromere in metaphase-arrested cells lacking microtubules, and decreased in the presence of tension (Fig. 3B). The pericentromeric levels of Sgo1-3A were not further increased by deletion of *RTS1*, indicating that PP2A-Rts1 controls Sgo1 levels at the pericentromere through a direct association (Fig. 3B). Furthermore, deletion of *RTS1* did not increase the levels of centromeric Bub1 (Supplemental Fig. S3C). We conclude that association with PP2A-Rts1 negatively regulates the pericentromeric localization of Sgo1.

### PP2A-Rts1 promotes timely release of Sgo1 from the pericentromere

If the interaction with PP2A-Rts1 is important for Sgo1 removal in the context of the cell cycle, we expected that Sgo1 dispersal into the nucleus would be delayed in *rts1Δ* or *sgo1-3A* cells. Wild-type and *rts1Δ* cells carrying *SGO1-yeGFP* and *MTW1-tdTomato* were released from a G1 arrest and imaged at 15-min intervals. We simultaneously analyzed a strain in which Sgo1-3A was tagged with GFP (*SGO1-3A-yeGFP*) and that also carried *MTW1-tdTomato* (Fig. 3C–E). Deletion of *RTS1* led to an ∼15-min delay in overall cell cycle progression, as judged by the splitting of Mtw1 foci (Fig. 3E). However, release of Sgo1 from the pericentromere was delayed by a further 15 min in *rts1Δ* cells (Fig. 3C). The Sgo1-3A protein showed a similar delay in release from the pericentromere (Fig. 3C), although overall cell cycle progression was not perturbed in this mutant (Fig. 3E). The 15-min delay in Sgo1 relocalization in *rts1Δ* and *sgo1-3A* cells was confirmed by scoring the number of time points in which pericentromeric Sgo1 was observed (Fig. 3D). We conclude that association with PP2A-Rts1 is required for the timely dissociation of Sgo1 from the pericentromere.

### Bub1 targets other than H2A-S121-P are important for Sgo1 removal under tension

**Figure 4.**
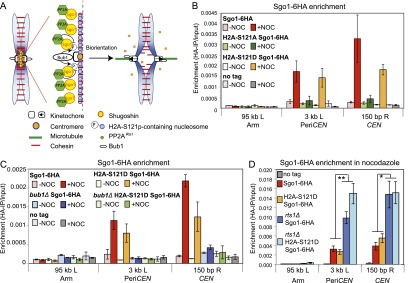
Bub1 substrates other than H2A-S121 are important for Sgo1 localization. (*A*) Hypothetical model for the regulation of Sgo1 localization by spindle tension. In the absence of tension, kinetochore-associated Bub1 phosphorylates chromatin-associated substrates, including H2A-S121, to create a binding site for Sgo1 in the pericentromere. Sgo1-bound PP2A^Rts1^ antagonizes these phosphorylations to release Sgo1 so that Sgo1 cycles on and off the pericentromere. In the presence of tension, Bub1 is moved away from the pericentromeric chromatin, and the pericentromeric binding site for Sgo1 is not maintained. (*B*) Dephosphorylation of H2A-S121 is not required for release of Sgo1 from the pericentromere. Wild type (AM10120), *H2A-S121A* (AM10128), and *H2A-S121D* (AM10137) carrying *SGO1-6HA* and *pMET3-CDC20* as well as a no tag control (AM2508) were arrested in metaphase with or without microtubules. The localization of Sgo1 was analyzed by ChIP-qPCR as described in Figure 1G. The mean of three experimental repeats is shown, with error bars representing standard error. (*C*) Bub1 is required for Sgo1 localization to the pericentromere in H2A-S121D cells. Wild-type (AM6390), *bub1Δ* (AM11962), *H2A-S121D* (AM10137), and *bub1Δ H2A-S121D* (AM11683) cells carrying *SGO1-6HA* and *pMET3-CDC20* as well as a no tag control (AM2508) were arrested in metaphase with or without microtubules, and the localization of Sgo1 was analyzed by ChIP-qPCR as described in Figure 1G. The mean of three experimental replicates is shown, with error bars representing standard error. (*D*) PP2A^Rts1^ affects Sgo1 levels independently of the phosphorylation status of H2A-S121. Wild-type (AM10123), *rts1Δ* (AM11977), *H2A-S121D* (AM10140), and *rts1Δ H2A-S121D* (AM11979) cells carrying *SGO1-6HA* as well as a no tag control (AM1176) were arrested in metaphase in the presence of nocodazole, and the localization of Sgo1 was analyzed by ChIP-qPCR at the indicated sites. Mean values of experimental replicates (*n* = *10* for AM1176, AM10123, AM11977; *n* = *7* for AM10140; *n* = *6* for AM11979) are shown, with error bars indicating standard error. The unpaired Student’s *t*-test was used to calculate significance. (**) *P* < 0.001; (*) *P* < 0.05.

Our findings suggest that the antagonistic activities of a kinetochore-localized kinase (Bub1) and a Sgo1-bound phosphatase (PP2A-Rts1) control Sgo1 localization in the pericentromere (Fig. 4A). Bub1 is known to phosphorylate histone H2A at residue S121, and this is important for Sgo1 association with the pericentromere (Fernius and Hardwick 2007; Kawashima et al. 2010; Haase et al. 2012). Since maintenance of Sgo1 at the pericentromere also requires Bub1 (Fig. 2H), we reasoned that dephosphorylation of H2A-S121 might be responsible for Sgo1 dispersal into the nucleus when sister kinetochores are under tension. Unfortunately, we were unable to monitor the phosphorylation status of H2A-S121 directly, as several attempts to raise antibodies to this site were not successful. As an alternative approach, we replaced S121 of H2A with aspartic acid to mimic the phosphorylated state. For comparison, we generated a phospho-null version by mutating S121 to alanine. The H2A-S121D (phospho-mimic) or H2A-S121A (phospho-null) alleles were introduced into cells carrying *pMET3-CDC20* and *SGO1-6HA* as the sole source of H2A, and the pericentromeric levels of Sgo1-6HA in metaphase-arrested cells were measured by ChIP-qPCR in the presence and absence of nocodazole. Figure 4B shows that the H2A-S121A mutation abolished Sgo1 localization at the pericentromere, as expected, confirming that phosphorylation at this residue is important for Sgo1 recruitment (Kawashima et al. 2010). Interestingly, cells carrying the H2A-S121D mutation behaved similarly to wild-type cells: Sgo1 was localized to the pericentromere only in the absence of spindle tension (Fig. 4B). Neither mutant affected total cellular levels of Sgo1 (Supplemental Fig. S4A). Therefore, the regulated dephosphorylation of H2A-S121 cannot be essential for Sgo1 removal from the pericentromere.

Next, we considered the possibility that H2A-S121 phosphorylation is not the only way that Bub1 promotes Sgo1 localization to the pericentromere. We deleted *BUB1* in cells where H2A-S121D is the only source of H2A and measured Sgo1 levels at the pericentromere in metaphase-arrested cells in both the presence and absence of spindle tension. Figure 4C shows that although H2A-S121D can support normal Sgo1 localization, this is dependent on Bub1. Again, cellular levels of Sgo1 were not affected (Supplemental Fig. S4B). Therefore, in addition to H2A-S121 phosphorylation, Bub1 plays other critical, as yet unknown, roles in promoting Sgo1 association with the pericentromere.

As a final test of the importance of regulating phosphorylation at residue S121 on H2A in controlling the different localization states of Sgo1, we examined the combined effect of H2A mutations and deletion of *RTS1.* If dephosphorylation of H2A contributes to Sgo1 removal, we would anticipate higher levels of Sgo1 at the pericentromere in the H2A-S121D mutant cells, but this is not the case (Fig. 4B). Moreover, deletion of *RTS1* led to an elevation of pericentromeric Sgo1 in H2A-S121D cells similar to that in wild-type cells, although total levels were not affected (Fig. 4D; Supplemental Fig. S4C). Therefore, like Bub1, PP2A-Rts1 exerts its effects on Sgo1 localization at the pericentromere in ways other than regulating H2A-S121 phosphorylation.

### Sgo1 removal from the pericentromere disengages the biorientation machinery

**Figure 5.**
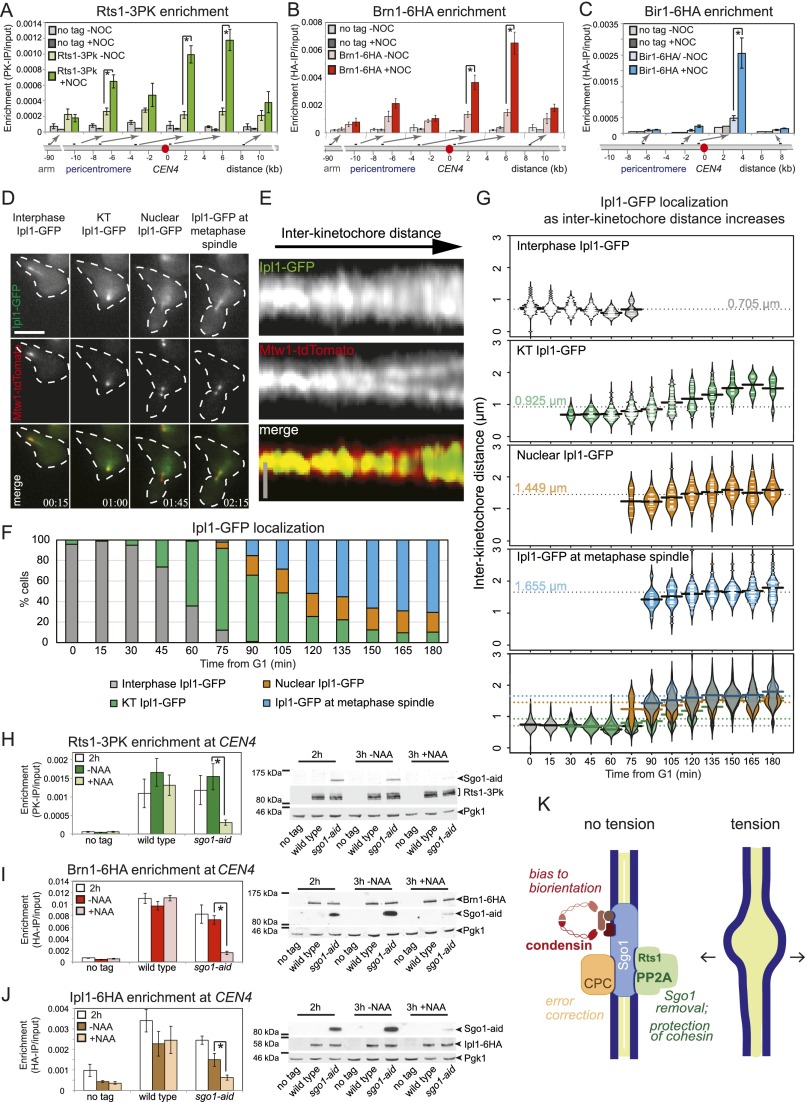
Sgo1 removal from the pericentromere leads to disassembly of the signaling platform that responds to a lack of tension at kinetochores. (*A–C*) Sgo1 effectors are removed from the centromere in response to intersister kinetochore tension. The association of PP2A^Rts1^ (*A*; Rts1), condensin (*B*; Brn1), and CPC (*C*; Bir1) subunits with the pericentromere is reduced in the presence of spindle tension. Strains carrying *pMET3-CDC20* and producing the indicated tagged proteins were arrested in metaphase with or without microtubules as described in Figure 1G, and the levels of the indicated proteins were examined by ChIP-qPCR using anti-PK (*A*) or anti-HA (*B*,*C*) antibodies and primer sets at the locations shown. Strains used were AM2508 (no tag), AM9639 (*RTS1-3PK*), AM8955 (*BRN1-6HA*), and AM6941 (*BIR1-6HA*). Mean values are given, and error bars represent standard error, except where *n* *=* *2* (no tag in *A*), where they represent range. In *A*, the number of experimental repeats was four (AM9639; *RTS1-*3PK) or two (AM2508, no tag). In *B*, data are shown from three experimental repeats for both no tag (AM2508) and *BRN1-6HA* (AM8955). In *C*, data are from three experimental replicates (AM6941; *BIR1-*6HA) or one experiment (AM2508; no tag). The unpaired Student’s *t*-test was used to calculate significance. (*) *P* < 0.05. (*D–G*) Ipl1 relocalizes from kinetochores during metaphase. Cells carrying *IPL1-yeGFP* and *MTW1-tdTomato* (strain AM9231) were imaged on a microfluidics device at 15-min intervals after release from G1 arrest. (*D*) Examples of Ipl1-GFP localization observed are shown. Time is given relative to release from G1. Bar, 5 μm. See also Supplemental Movie S3. (*E*) Line scans across kinetochore foci of single cells were assembled from 100 images to generate a V plot showing Ipl1-GFP localization as interkinetochore distance increases. Bar, 2 μm. (*F*) Bar chart with the fraction of cells with the indicated Ipl1 localization at each time point is shown. (*G*) The distance between Mtw1-tdTomato signals and the localization of Ipl1-yeGFP was scored in at least 77 cells for each time point. The bean plot shows the distribution of interkinetochore distances for which each localization type was scored. Lines within the beans represent individual cells. Beans for small sets of cells (*N* < 6) are not shown. The horizontal line represents the mean. (*H–J*) Sgo1 is required for the maintenance of PP2A^Rts1^, condensin, and the CPC at the centromere. Wild-type and *sgo1-aid* strains carrying *RTS1-3PK* (*H*), *BRN1-6HA* (*I*), or *IPL1-6HA* (*J*) and a no tag control were arrested in metaphase by treatment with nocodazole for 2 h, and one-third of the culture was harvested. The remaining culture was split, half was treated with NAA to induce Sgo1-aid degradation, and both treated and untreated cultures were harvested after a further 1 h in the presence of nocodazole. Anti-aid, anti-Pgk1, and anti-PK (*H*) or anti-HA (*I*,*J*) immunoblots are shown to confirm Sgo1-aid degradation. Pgk1 is shown as a loading control. Also shown are the mean results of qPCR after anti-PK (*H*) or anti-HA ChIP (*I*,*J*) from four experimental replicates, with error bars representing standard error. The two-tailed paired Student’s *t*-test was used to calculate significance. (*) *P* < 0.05. (*K*) Schematic diagram summarizing disassembly of the pericentromeric signaling platform.

During mitosis, Sgo1 engages Ipl1 and condensin to promote chromosome biorientation (Verzijlbergen et al. 2014). Importantly, once biorientation is established, the error correction machinery must be deactivated, presumably in a chromosome-autonomous manner. We reasoned that Sgo1 removal from the pericentromere could contribute to this chromosome-autonomous response to tension by triggering dissociation of its effectors from the pericentromere. Indeed, we found that the pericentromeric association of the PP2A regulatory subunit Rts1, the condensin component Brn1, and the CPC subunit Bir1 (which depends on Sgo1 for its maintenance at the centromere) (Supplemental Fig. S5A) were all negatively regulated by tension. Centromeric ChIP-qPCR values were reduced more than fourfold in the presence, compared with the absence, of tension for all three proteins (Fig. 5A–C). This suggests that disassembly of the pericentromeric platform of Sgo1 leads to the dispersal of its effector proteins from this region.

To analyze the tension dependence of Aurora B/Ipl1 localization in more detail, we imaged live cells producing Ipl1-GFP as they progressed from G1 into a metaphase arrest induced by depletion of *CDC20* (Fig. 5D–G). Ipl1 relocalization onto the spindle during anaphase is well documented; however, kinetochore, nuclear, and spindle localizations have all been observed in metaphase, and the relative timing of these localizations has been unclear (Tanaka et al. 2002; Buvelot et al. 2003; Pereira and Schiebel 2003; Woodruff et al. 2010; Nakajima et al. 2011; Zimniak et al. 2012). We found that soon after release from G1, Ipl1-GFP coalesced from its interphase localization on microtubules into a bright dot that colocalized with the kinetochores. As Mtw1-tdTomato foci split, Ipl1-GFP lost its kinetochore localization and was briefly released into the nucleus before associating with the metaphase spindle (Fig. 4D–F; Supplemental Movie S3). Importantly, the average distance occupied by kinetochores decorated by Ipl1-GFP (0.925 μm) (Fig. 5G) correlates with the average distance at which Sgo1-GFP is localized at the pericentromere (1.047 μm) (Fig. 1F), while the other types of localization occur at longer interkinetochore distances. Therefore, like Sgo1, Ipl1 shows tension-dependent removal from kinetochores.

Next, we asked whether Sgo1 removal from the pericentromere is sufficient to relocate the CPC, condensin, and PP2A from this region. We generated an auxin-inducible degron version of Sgo1 to enable artificial removal of Sgo1 from the pericentromere in cells arrested in mitosis. Wild-type or *sgo1-aid* cells carrying tagged PP2A (*RTS1-3PK*), condensin (*BRN1-6HA*), or CPC (*BIR1-6HA*, *IPL1-6HA*) components were arrested in metaphase by treatment with nocodazole, and the levels of the tagged proteins at *CEN4* were measured by ChIP-qPCR. Subsequently, we treated half the culture with NAA (to induce Sgo1 degradation), while the other half received no treatment (Fig. 5H–J; Supplemental Fig. S5B). After a further 1 h, the levels of the proteins at *CEN4* were again measured by ChIP-qPCR. In all cases, NAA treatment induced degradation of Sgo1 in metaphase and led to almost complete removal of the effector proteins from the pericentromere, while in untreated cells, Sgo1 was maintained, and the localization of its effector proteins persisted (Fig. 5H–J; Supplemental Fig. S5B). We conclude that Sgo1 removal from the pericentromere in metaphase is sufficient for the release of condensin, CPC, and PP2A-Rts1 from this region (Fig. 5K).

### Tethered Sgo1 is sufficient to maintain Aurora B at the centromere in the presence of microtubules

**Figure 6.**
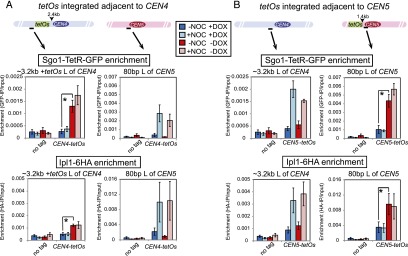
Sgo1 removal from the pericentromere upon biorientation is required for Aurora B (Ipl1) dissociation. (*A*,*B*) Tethered Sgo1 is sufficient to retain Ipl1 at the centromere in the presence of spindle tension. Strains carrying *SGO1-tetR-GFP*, *IPL1-6HA*, and *pMET3-CDC20* and with *tetO* repeats integrated ∼2.4 kb to the left of *CEN4* (AM12151; *A*) or ∼80 bp to the left of *CEN5* (AM12148; *B*) were released from a G1 arrest into medium containing methionine to induce a metaphase arrest either with or without nocodazole and in both the presence (+DOX) and absence (−DOX) of doxycycline. Anti-GFP (*top* graphs) and anti-HA (*bottom* graphs) ChIP was performed, and samples were analyzed by qPCR with primers specific to the indicated sites. A no tag strain (AM2508) was also analyzed, and data are reproduced in *A* and *B*. The mean values from four experimental replicates are shown, with error bars representing standard error. The two-tailed paired Student’s *t*-test was used to calculate significance. (*) *P* < 0.05.

If removal of the CPC from the pericentromere upon biorientation is triggered by tension-dependent dissociation of Sgo1, we reasoned that Sgo1 tethered to the pericentromere would prevent CPC removal even when sister kinetochores should be under tension. We integrated *tetO* arrays adjacent to the centromere of chromosome IV (Fig. 6A) or chromosome V (Fig. 6B) at sites that are known to separate when sister kinetochores are under tension (He et al. 2000; Tanaka et al. 2000) and expressed Sgo1-TetR-GFP in cells also carrying *IPL1-6HA*. These cells were arrested in metaphase by depletion of *CDC20* (sister kinetochores are under tension) with or without nocodazole and in either the presence (+DOX; no Sgo1-TetR-GFP tethering) or absence (−DOX; Sgo1-TetR-GFP bound to *tetO*s) of doxycycline. We first confirmed that tethered Sgo1-TetR-GFP remained bound to *tetO* repeats as they separate under tension. In metaphase-arrested cells with *tetO* repeats adjacent to *CEN4*, similar levels of Sgo1-TetR-GFP associated with a site close to *CEN4* in the presence and absence of nocodazole (Fig. 6A, top left graph); however, Sgo1-TetR-GFP close to *CEN5* was removed in the presence of tension (Fig. 6A, top right graph). Conversely, in cells where *tetO* repeats were close to *CEN5*, Sgo1-TetR-GFP remained associated with a site near to *CEN5* in the presence of tension (Fig. 6B, top right graph) but not a site near to *CEN4* (Fig. 6B, top left graph). Importantly, Ipl1-6HA localization was significantly increased adjacent to Sgo1-TetR-GFP tethered to either *CEN4* or *CEN5* but not at the site close to the centromere lacking the tether (Fig. 6A,B, bottom panels). (Note that integration of *tetO*s at either *CEN4* or *CEN5* prevented recruitment of normal levels of Ipl1 to adjacent sites in the absence of Sgo1-TetR-GFP tethering for reasons that are currently unclear [Fig. 6A,B, +DOX condition].) Therefore, the dissociation of Ipl1 in metaphase requires Sgo1 release from the pericentromere. Overall, our results support a model in which tension-triggered Sgo1 removal leads to disassembly of the pericentromeric platform that governs error correction (Supplemental Fig. S6).

### Suppression of sister kinetochore biorientation ensures the retention of pericentromeric Sgo1 during meiosis I

**Figure 7.**
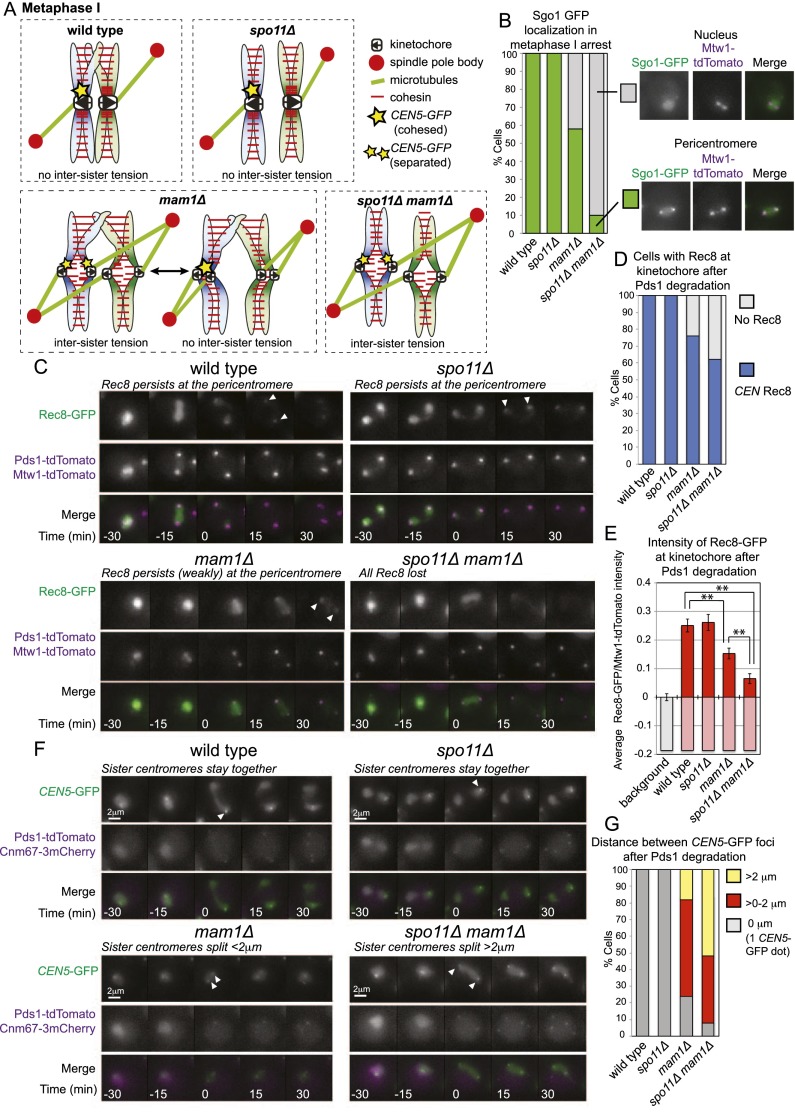
Sister kinetochore tension leads to partial deprotection of cohesin in meiosis I. (*A*) Schematic diagram showing possible kinetochore orientations at meiosis I for the indicated genotypes. (*B*) Sgo1 is released from the pericentromere upon kinetochore biorientation during meiosis I. Wild-type (AM15137), *spo11Δ* (AM15139), *mam1Δ* (AM15138), and *spo11Δ mam1Δ* (AM15140) cells carrying *SGO1-yeGFP MTW1-tdTomato* and *pCLB2-CDC20* were induced to sporulate, transferred to a microfluidics device after 4 h, and imaged every 15 min. The area occupied by Sgo1-yeGFP was scored in 50 cells in the first frame after Mtw1-tdTomato kinetochore foci split and categorized as pericentromere (foci covering <2 μm^2^) or dispersed nuclear localization (no distinct foci, but signal of at least three times the intensity of the background signal over >2 μm^2^). Example images are shown. (*C–E*) Reduced Rec8 at centromeres during anaphase I in *mam1Δ* and *spo11Δ mam1Δ* cells. Wild-type (AM13716), *spo11Δ* (AM13718), *mam1Δ* (AM13717), and *spo11Δ mam1Δ* (AM13719) cells carrying *REC8-GFP*, *MTW1-dtTomato*, and *PDS1-tdTomato* were resuspended in sporulation medium for 2 h before loading onto a microfluidics plate and imaged at 15-min intervals. (*C*) Example sequences are shown, with time shown relative to the first frame in which Pds1 degradation has occurred (*t* = 0, anaphase I). Arrowheads indicate centromeric Rec8. (*D*) The percentage of cells in which Rec8-GFP colocalized with Mtw1-tdTomato kinetochore foci in the first or second time frame after Pds1 degradation (*t* = 15 or 30) is given after scoring the behavior of 50 cells. (*E*) The average intensity of Rec8-GFP signal was measured in the area occupied by and between the Mtw1-tdTomato signal for each cell. The average ratio of Rec8-GFP/Mtw1-tdTomato intensity is given for 50 cells. As a measure of background fluorescence, we analyzed kinetochore clusters of wild-type cells in anaphase II, where all Rec8 would be expected to be lost. Error bars represent standard error. The unpaired Student’s *t*-test was used to calculate significance. (**) *P* < 0.001. (*F*,*G*) Sister chromatids segregate at meiosis I in a fraction of *mam1Δ* and *spo11Δ mam1Δ* cells, indicating precocious loss of pericentromeric cohesion. Wild type (AM13431), *spo11Δ* (AM13979), *mam1Δ* (AM13978), and *spo11Δ mam1Δ* (AM13980) with *tetO* repeats integrated at *CEN5* of one homolog expressing *tetR-GFP* and carrying *CNM67-3mCherry* and *PDS1-tdTomato* were resuspended in sporulation medium for 2 h before loading onto a microfluidics plate and imaging at 15-min intervals. (*F*) Representative sequences are shown. Times are given relative to Pds1 degradation (*t* = *0*). Arrowheads indicate *CENV-GFP* foci. (*G*) The greatest distance between sister *CENV-GFP* foci was measured after Pds1 degradation but before SPB reduplication for 50 cells.

During meiosis I, sister kinetochores must be mono-oriented (attached to microtubules from the same pole), and therefore the biorientation of sister kinetochores is suppressed. Inactivation of the monopolin complex, which is required for kinetochore mono-orientation, does not abolish the protection of pericentromeric cohesion during meiosis I, which has led to the idea that a lack of tension between sister kinetochores during meiosis I is not important for the maintenance of Sgo1 (Toth et al. 2000; Rabitsch et al. 2003; Petronczki et al. 2006; Matos et al. 2008). However, chromosomes have the opportunity to attach to the spindle in a variety of orientations in monopolin mutants due to the presence of chiasmata that provide resistance to spindle forces (Fig. 7A), so it is likely that not all sister kinetochores are bioriented. As a measure of sister kinetochore biorientation in cells lacking monopolin, we examined the separation of TetR-GFP foci bound to *CEN5-*proximal *tetO* repeats in cells arrested in metaphase I by depletion of *CDC20* (by placement under the control of the mitosis-specific promoter *pCLB2*) (Lee and Amon 2003). In wild-type cells, since sister kinetochore biorientation is suppressed, a single GFP focus is observed (Supplemental Fig. S7A). In cells lacking the monopolin component Mam1, separated *CEN5*-GFP foci were observed in ∼30% of cells (Supplemental Fig. S7A). While this indicates that mono-orientation is defective in *mam1Δ* cells, the fraction of cells with separated *CEN5-*GFP foci is much lower than expected if sister kinetochores on chromosome V were bioriented in all cells. We reduced the number of ways that kinetochores could stably attach to microtubules in metaphase I by deleting *SPO11*, the endonuclease required for the initiation of meiotic recombination, thereby abolishing chiasmata (Fig. 7A; Keeney et al. 1997; Shonn et al. 2000). In *spo11Δ mam1Δ* cells, the percentage of cells with separated *CEN5-GFP* was increased to ∼60%, indicating that eliminating chiasmata facilitates sister kinetochore biorientation in *mam1Δ* cells (Supplemental Fig. S7A).

The increased sister kinetochore biorientation of *mam1Δ spo11Δ* cells gave us the opportunity to test how tension across sister kinetochores influences Sgo1 association with the pericentromere during meiosis I. Wild-type, *spo11Δ*, *mam1Δ*, and *spo11Δ mam1Δ* cells were arrested in metaphase I by depletion of *CDC20* (*pCLB2-CDC20*), and the area occupied by Sgo1-GFP was measured in live cells directly after kinetochore clusters became bilobed (Fig. 7B). Although Sgo1-GFP formed pericentromeric foci in all wild-type and *spo11Δ* cells, only diffuse nuclear fluorescence was observed in 42% of *mam1Δ* cells and 88% of *spo11Δ mam1Δ* cells (Fig. 7B). Therefore, Sgo1 localization is responsive to kinetochore orientation in meiosis I too. We confirmed these observations by ChIP-qPCR: Centromeric Sgo1 levels were lowest in *spo11Δ mam1Δ* cells in which sister kinetochore biorientation is most frequent (Supplemental Fig. S7B). Interestingly, centromeric Sgo1 levels were highest in *spo11Δ* cells in which both intersister tension and interhomolog tension are abolished (Fig. 7A; Supplemental Fig. S7B). Overall centromeric Sgo1 levels in *mam1Δ* cells were comparable with wild-type cells (Supplemental Fig. S7B), perhaps representing the average of a population of cells that includes attachments that lack tension as well as those that generate intersister tension (Fig. 7A). Expression of the mitotic cyclin *CLB3* in meiosis I causes an albeit milder defect in sister kinetochore mono-orientation than *mam1Δ* without affecting overall centromeric levels of Sgo1 (Miller et al. 2012). Similar to our observations with *mam1Δ* cells, we found that deletion of *SPO11* both increases sister kinetochore biorientation and decreases Sgo1 levels at centromeres in *pCUP1-CLB3* cells (Supplemental Fig. S7C,D). The idea that Sgo1 levels at the pericentromere are sensitive to all types of tension at kinetochores was confirmed by treatment of wild-type and *spo11Δ* metaphase I-arrested cells with the microtubule-destabilizing drug benomyl, which resulted in thin metaphase I spindles and increased levels of Sgo1 at the pericentromere (Supplemental Fig. S7E). Together, these findings indicate that the pericentromeric levels of Sgo1 are responsive to spindle tension also during meiosis I and that Sgo1 levels at the pericentromere are lowest when sister kinetochores are bioriented.

### Sister kinetochore biorientation in meiosis I leads to partial deprotection of cohesin

The reduced pericentromeric Sgo1 in metaphase I-arrested *spo11Δ mam1Δ* cells implied that cohesin may not be efficiently protected in these cells. Consistent with this idea, *spo11Δ mam1Δ* cells undergo a single meiotic division in which sister chromatids separate to opposite poles (Matos et al. 2008). We examined the localization of the meiotic cohesin subunit Rec8 on spread meiotic chromosomes from cells progressing synchronously through meiosis after release from a prophase I arrest (Carlile and Amon 2008). Compared with wild-type, *spo11Δ*, or *mam1Δ* cells, the fraction of cells with Rec8 only in the vicinity of centromeres (as identified by costaining the kinetochore subunit Ndc10) was reduced in *spo11Δ mam1Δ* cells (Supplemental Fig. S7F–I). We confirmed these observations in live single cells progressing through meiosis by examining Rec8-GFP localization immediately after Pds1-tdTomato (securin) degradation in cells that also carried Mtw1-tdTomato (to label kinetochores) (Fig. 7C,D). Interestingly, although 100% of wild-type and *spo11Δ* cells retained Rec8 at kinetochores, Rec8 was undetectable at kinetochores in 24% of *mam1Δ* and 38% of *spo11Δ mam1Δ* cells directly after separase activation in meiosis I (Fig. 7D). Since our findings above suggest that chromosomes can attach to the spindle in a variety of orientations in *mam1Δ* cells and, to a lesser extent, *spo11Δ mam1Δ* cells, it is likely that not all chromosomes within each cell behave in a uniform manner. Therefore, we used fluorescence intensity measurements to quantify the average Rec8-GFP signal remaining at kinetochore clusters directly after Pds1 degradation and expressed this as a ratio of the Mtw1-tdTomato signal (Fig. 7E). These measurements confirmed a significant overall reduction in Rec8 levels at centromeres during anaphase I in *mam1Δ* cells and a further reduction in *spo11Δ mam1Δ* cells. This is consistent with the idea that biorientation of sister chromosomes during metaphase I impairs the maintenance of pericentromeric Rec8 during anaphase I.

To determine whether the reduced pericentromeric Rec8 in *mam1Δ* and *spo11Δ mam1Δ* mutants results in the segregation of sister chromosomes to opposite poles in meiosis I, we filmed cells carrying *CEN5-GFP* foci on one homolog together with Pds1-tdTomato and the spindle pole body marker Cnm67-3mCherry (Fig. 7F). We scored the percentage of cells in which *CEN5-GFP* segregated away from each other (*CEN5-GFP* foci separated to >2 μm) directly following Pds1 degradation in meiosis I. Separation of sister *CEN5-GFP* foci to a distance of <2 μm suggests that sister kinetochores are bioriented, but pericentromeric cohesion is retained. As reported previously for *mam1Δ* mutants (Toth et al. 2000), in a large fraction (58%) of cells, *CEN5*-GFP separated only a short distance (<2 μm), indicating sister kinetochore biorientation without loss of cohesion, and this phenotype was also apparent in 40% of *spo11Δ mam1Δ* cells (Fig. 7G). This indicates that biorientation of individual kinetochores may not in itself be sufficient for sister centromeres to segregate to opposite poles. Remarkably, however, 18% of *mam1Δ* cells and 52% of *spo11Δ mam1Δ* cells segregated sister *CEN5-*GFP foci to opposite poles immediately following Pds1 degradation in meiosis I, indicating a failure to protect pericentromeric cohesion (Fig. 7G). These results indicate that suppression of sister kinetochore biorientation during meiosis I is required to ensure the proper protection of pericentromeric cohesion, likely through maintaining the localization of Sgo1.

## Discussion

### Shugoshin: the tension sensor

Ever since Nicklas’ elegant micromanipulation experiments (Nicklas and Koch 1969) showed that tension across centromeres stabilizes kinetochore attachments, the mechanistic basis of this stabilization has been pondered. More recent evidence has suggested that tension stabilizes attachments both directly (Akiyoshi et al. 2010) and, through opposition of the destabilizing kinase Aurora B, indirectly (Lampson and Cheeseman 2011). However, it has remained unclear how the state of tension at sister kinetochores is read so that the response to a lack of tension can be silenced. Tension-dependent changes in shugoshin localization have been observed in mouse spermatocytes and oocytes and human somatic cells (Gomez et al. 2007; Lee et al. 2008; Liu et al. 2013a). In these systems, shugoshin relocates from the inner centromere to the kinetochore once sister kinetochore biorientation is established. Similarly, here, we showed that budding yeast shugoshin associates with the pericentromere only when sister kinetochores are not under tension. Moreover, we provided evidence that the crux of the response to sister kinetochore biorientation is the tension-dependent removal of shugoshin from the pericentromere.

Shugoshin fits all of the criteria for the fundamental tension sensor. First, shugoshin associates with the pericentromere only when sister kinetochores are not under tension. Second, shugoshin can reversibly associate with the pericentromere during prometaphase and metaphase where kinetochore-microtubule interactions are perturbed. Third, the pericentromeric localization of the tension-sensing machinery depends on shugoshin. Fourth, the tension-dependent localization of shugoshin to the pericentromere is chromosome-autonomous. Fifth, shugoshin is irreversibly destroyed when the commitment to chromosome segregation is made in anaphase.

We propose that shugoshin removal from the pericentromere in mitotic metaphase signals sister kinetochore biorientation and initiates the transition to anaphase. Dispersal of shugoshin abolishes the platform for Aurora B at the pericentromere, thereby disengaging the error correction machinery and reinforcing kinetochore–microtubule attachments. This will in turn suppress SAC signaling from unattached kinetochores, ultimately allowing loss of cohesion and chromosome segregation. However, Sgo1 dispersal cannot be the only mechanism by which Ipl1 is inactivated in response to tension. Truncation of the CPC component Sli15 allows Ipl1 clustering on microtubules and overrides the requirement for its Sgo1-dependent centromeric targeting, yet chromosomes biorient normally (Campbell and Desai 2013), suggesting that additional factors are able to counteract Ipl1 activity upon tension establishment.

### The importance of suppressing sister kinetochore biorientation during meiosis I

In contrast to mitosis, during meiosis I, sister kinetochores are mono-oriented. It has been suggested that the suppression of sister kinetochore biorientation in meiosis I ensures the protection of pericentromeric cohesin (Vaur et al. 2005; Gomez et al. 2007; Lee et al. 2008). Fission yeast cells defective in sister kinetochore mono-orientation fail to properly protect pericentromeric cohesin (Vaur et al. 2005; Yokobayashi and Watanabe 2005). This was not initially thought to be the case in budding yeast, as monopolin mutants retain pericentromeric Rec8 during anaphase I, and sister chromatids remain cohesed after securin degradation in meiosis I (Toth et al. 2000). However, our data indicate that sister kinetochore biorientation is not complete during meiosis I in monopolin mutants. By additionally removing chiasmata, we were able to increase the frequency of cells with sister kinetochores under tension. Analysis of cells lacking monopolin and chiasmata showed that the suppression of sister kinetochore biorientation during meiosis I helps to retain shugoshin at the pericentromere and contributes to the maintenance of pericentromeric cohesion during meiosis I. This indicates that the state of sister kinetochore tension may play a role in ensuring the step-wise loss of cohesin in meiosis through controlling shugoshin localization. However, it is unlikely that tension between sister kinetochores is sufficient for the deprotection of cohesion, and other mechanisms must contribute. In a considerable fraction of cells lacking both monopolin and chiasmata, sister kinetochore biorientation is achieved, yet sister chromatids fail to segregate to opposite poles following securin degradation during meiosis I, indicating that pericentromeric cohesion persists (Fig. 7; Toth et al. 2000; Matos et al. 2008). In contrast, inactivation of *SGO1* in monopolin mutant cells allows nuclear division without a delay, and *spo11Δ mam1Δ* cells lacking Sgo1 segregate sister chromatids to opposite poles during meiosis I (Katis et al. 2004; Petronczki et al. 2006; Kiburz et al. 2008). This suggests that even when sister kinetochores are under tension, a low level of Sgo1 persists at some pericentromeres and that this is sufficient for cohesin protection. Alternatively, these observations raise the possibility that once cohesin protection is in place, events downstream from Sgo1 removal are required to reverse it. While we cannot currently distinguish between these models, these observations demonstrate that sister kinetochore biorientation is unlikely to be sufficient for the deprotection of cohesion, and additional mechanisms must contribute. Indeed, in mouse oocytes, the PP2A inhibitor I2PP2A/Set1β colocalizes with Rec8 only in meiosis II, and its depletion prevents sister chromatid segregation during meiosis II (Chambon et al. 2013). Therefore, although suppression of sister chromatid biorientation facilitates the maintenance of pericentromeric cohesion during meiosis I, its deprotection during meiosis II is likely to require additional factors.

### Opposing kinases and phosphatases trigger shugoshin redistribution under tension

What are the molecular events that lead to Sgo1 redistribution? Although the detailed tension-dependent mechanism is yet to be worked out, it is clear that dephosphorylation is key to this process (Supplemental Fig. S6). We showed that PP2A-Rts1 negatively regulates Sgo1 levels at the centromere. We propose that Sgo1-bound PP2A, and possibly other phosphatases too, promote dephosphorylation of as yet unknown chromatin-associated substrates, the phosphorylation of which is required for Sgo1 association with the pericentromere. In the absence of tension, Sgo1 remains pericentromere-bound because of the proximity of the kinetochore-bound kinase Bub1. Spindle tension leads to the spatial separation of Bub1 from the chromatin, leading to the reversal of phosphorylation of its chromatin-bound substrates by PP2A-Rts1, releasing Sgo1. Eventually, upon stable biorientation, Bub1 kinase itself dissociates from its Spc105/Spc7/KNL1 receptor in the kinetochore due to reversal of Mps1-dependent phosphorylation by PP1, which also binds to Spc105/Spc7/KNL1 (Pinsky et al. 2009; Vanoosthuyse and Hardwick 2009; Meadows et al. 2011; Rosenberg et al. 2011; Espeut et al. 2012; London et al. 2012; Shepperd et al. 2012; Yamagishi et al. 2012). We speculate that moving kinetochores away from the reach of Aurora B, which is known to antagonize PP1 (Pinsky et al. 2006a; Liu et al. 2010; Rosenberg et al. 2011), will be key for Bub1 dissociation from the kinetochore (Funabiki and Wynne 2013). This reciprocal kinetochore–pericentromere phosphorylation model provides an attractive framework for sensing interkinetochore tension and raises additional questions for future studies. Interestingly, a recent report in somatic cells showed that reversal of the CDK-dependent phosphorylation of shugoshin triggers its relocation onto the Bub1-dependent phospho-H2A receptor in the kinetochore (Liu et al. 2013a). This suggests that shugoshins might undergo phospho-regulation by multiple kinases. Furthermore, the spectrum of phospho-regulated substrates is likely to be broad and, at a minimum, include shugoshin itself and histones (Kawashima et al. 2010; Liu et al. 2013b; Ng et al. 2013). While H2A-S121-P is required for Sgo1 localization within the pericentromere, we found that regulated phosphorylation of this site does not underlie Sgo1 behavior in response to tension. Unraveling the important enzymes and substrates in the phospho-regulation of shugoshin will be an important priority for the future. Shugoshins have been found to be misregulated in human cancers. This suggests that exquisite control of this fundamental tension sensor is likely to be essential in protecting against aneuploidy and its associated diseases.

## Materials and methods

### Yeast strains and plasmids

All yeast strains were derivatives of W303 or SK1 and are listed in Supplemental Table S1. *SCC1-6HA* was described in Megee and Koshland (1999). A PCR-based approach was used to tag Bub1, Mtw1, Bir1, and Ndc10 with 6HA; Mtw1 with tdTomato; replace the *CLB3* promoter with *pCUP1*; and generate null alleles (Longtine et al. 1998; Knop et al. 1999). *SGO1-yeGFP*, *BUB1-yeGFP*, and *IPL1-yeGFP* were also generated by PCR-based epitope tagging (Sheff and Thorn 2004). Auxin-inducible degron tagging was performed as described (Nishimura et al. 2009). *pMET3-CDC20* was described in Clift et al. (2009). *SGO1-6HA*, *IPL1-6HA*, *BRN1-6HA*, *RTS1-3PK*, and *SGO1-TetR-GFP* were described in Verzijlbergen et al. (2014). The *ipl1-as5* and *stu2-277* alleles were described in Pinsky et al. (2006b) and He et al. (2001), respectively. *pCLB2-CDC20* was described in Lee and Amon (2003). *REC8-13Myc* and *SGO1-9Myc* were described in Marston et al. (2004). *REC8-GFP*, *MTW1-tdTomato*, *PDS1-tdTomato*, and *CNM67-3mCherry* were described in Matos et al. (2008). *CEN5-GFP* and *NDC10-6HA* were described in Toth et al. (2000). To label chromosome III close to the centromere with GFP, a ∼700-base-pair (bp) fragment adjacent to *CEN3* was cloned into *pRS306-112xtetO* (Michaelis et al. 1997) to generate plasmid AMp679, which was integrated in a strain producing TetR-GFP. Plasmid pER1 (*CEN6-TRP1-HTA1-HTB1*) was a kind gift from Dr. F. van Leeuwen (Netherlands Cancer Institute). Plasmids AMp920 (*H2A-S121D*) and AMp921 (*H2A-S121A*) were generated by site-directed mutagenesis of pER1 using a QuikChange II XL kit (Agilent Technologies).

### Growth conditions

To arrest cells in metaphase by Cdc20 depletion, strains carrying *pMET3-CDC20* were arrested in G1 in synthetic medium lacking methionine (SC/−Met/D) with α factor (4 or 5 mg/mL). Cells were then washed with rich medium lacking glucose (YEP) and released into rich medium containing 8 mM methionine (YPDA/Met). Methionine was readded to 4 mM every hour. To achieve a metaphase arrest in the absence of microtubules, 15 μg/mL nocodazole was added immediately after release into YPDA/Met and readded to 7.5 μg/mL every hour. To inhibit Ipl1-as5, 1 NA-PP1 was added to a final concentration of 50 mM. The *stu2-277* allele was inactivated by shifting to 37°C. Doxycycline was used at 5 μg/mL. Meiosis was performed as described in Marston et al. (2003). For meiotic prophase I block–release experiments using strains carrying *pGAL-NDT80* and *GAL4-ER*, prophase release was induced by addition of β-estradiol to 1 mM (Carlile and Amon 2008). Benomyl was added to 90 μg/mL 30 min before harvesting. Copper sulfate was used at 50 μM.

### Immunofluorescence

Indirect immunofluorescence was performed as described in Visintin et al. (1999). Tubulin was visualized using a rat anti-tubulin antibody (AbD Serotec) at a dilution of 1:50 and an anti-rat FITC-conjugated antibody (Jackson ImmunoResearch) at a dilution of 1:100. For detection of Sgo1-6HA, a mouse HA.11 antibody (Covance) at a dilution of 1:500 and an anti-mouse Cy3-conjugated antibody (Jackson ImmunoResearch) at a dilution of 1:100 were used. Chromosome spreads were performed as described in Bizzari and Marston (2011).

### Western blotting

Samples were prepared for Western blotting as described in Clift et al. (2009) except that some antibodies were detected using the fluorescence-based Li-Cor Odyssey system. Antibodies used were mouse anti-HA 12CA5 (Roche), mouse anti-PK(V5) (AbD Serotec), mouse anti-aid (Cosmo Bio Co.), and rabbit or mouse anti-Pgk1 (laboratory stock and Life Technologies, respectively).

### ChIP

ChIP was performed as described in Fernius et al. (2013) using mouse anti-HA (12CA5, Roche Diagnostics), mouse anti-PK(V5) (AbD Serotec), or rabbit anti-GFP (a kind gift of Dr. Eric Schirmer, University of Edinburgh) antibodies. For experiments in Supplemental Figure S2, A–C, qPCR was performed using a Bio-Rad iCycler machine and the protocol described in Fernius and Marston (2009). For all other experiments shown, qPCR was performed on a Roche LightCycler.

### Microscopy methods

Fluorescent microscopy analysis of fixed cells was performed using a Zeiss Axioplan 2 microscope. Images were taken using a Hamamatsu camera operated through Axiovision software and processed using ImageJ software (National Institutes of Health).

For live-cell imaging, the ONIX microfluidic perfusion platform by CellASIC was used within a heated chamber set to 30°C, with the exception of the experiment shown in Figure 1, E and F, where an Attofluor (Life Technologies) chamber heated to 25°C was used. The microfluidics system was set up on a DeltaVision Core system with an Olympus IX-71 microscope with ultimate focus, and a 100× Plan Apochromat/1.4 NA (oil) lens was used for taking images. For imaging vegetative cells, G1-arrested cells were loaded onto the plate, and we began imaging (15-min intervals) immediately upon release from the arrest; six to eight Z-sections 0.6–0.7 μm apart were taken for each field, with the exception of the experiment shown in Supplemental Figure S1I, where cycling cells were loaded onto the plate and filmed as above. For imaging of meiotic samples, cells were induced to sporulate by resuspension in sporulation medium in flasks for 2.5 h before transferring to a microfluidics plate, and we began imaging ∼1 h later at 15-min intervals. For each image, six Z-sections 1 μm apart were grabbed at 10% T for the green channel and 5% T for the red channel with exposure times of 0.3 sec (Rec8-GFP), 0.2 sec (*CENV*-GFP), and 0.2 sec (red channel). ONIX software was used to control the microfluidics system, and SoftWoRx software was used for the control of the DeltaVision microscopy system and taking images. Image analysis was performed using Image-Pro and ImageJ programs, and final images were assembled using Adobe Photoshop. A custom-written plug in for Image J was used to generate V plots. Line scans were manually drawn across Mtw1-tdTomato kinetochore foci/focus of 100 single cell images. The center point between the two brightest pixels was chosen as a reference for alignment, and line scans were ordered according to their length. Details are available on request.

For fluorescence intensity measurements of kinetochore/microtubule-associated Sgo1-GFP signal, we used the “box in box” method described in Hoffman et al. (2001), with the modification that two ellipses were used, as this allowed better isolation of the kinetochore and spindle area from the nuclear area (see Supplemental Fig. S1J).
